# Robotic Stereotactic Radiosurgery in Melanoma Patients with Brain Metastases under Simultaneous Anti-PD-1 Treatment

**DOI:** 10.3390/ijms19092653

**Published:** 2018-09-07

**Authors:** Maike Trommer-Nestler, Simone Marnitz, Martin Kocher, Daniel Rueß, Max Schlaak, Sebastian Theurich, Michael von Bergwelt-Baildon, Janis Morgenthaler, Karolina Jablonska, Eren Celik, Maximilian I. Ruge, Christian Baues

**Affiliations:** 1 Department of Radiation Oncology and Cyberknife Center, University Hospital of Cologne, Kerpener Str. 62, 50937 Cologne, Germany; simone.marnitz-schulze@uk-koeln.de (S.M.); janis.morgenthaler@uk-koeln.de (J.M.); karolina.jablonska@uk-koeln.de (K.J.); eren.celik@uk-koeln.de (E.C.); christian.baues@uk-koeln.de (C.B.); 2 Center for Integrated Oncology (CIO Köln Bonn), University of Cologne, 50937 Cologne, Germany; martin.kocher@uk-koeln.de (M.K.); daniel.ruess@uk-koeln.de (D.R.); maximilian.ruge@uk-koeln.de (M.I.R.); 3 Radio Immune-Oncology Consortium (RIO), University Hospital of Cologne, 50937 Cologne, Germany; Max.Schlaak@med.uni-muenchen.de (M.S.); Sebastian.Theurich@med.uni-muenchen.de (S.T.); Michael.Bergwelt@med.uni-muenchen.de (M.v.B.-B.); 4 Department of Stereotaxy and Functional Neurosurgery, Centre of Neurosurgery, University Hospital of Cologne, 50937 Cologne, Germany; 5 Institute for Neuroscience and Medicine, Research Center Juelich, Wilhelm-Johnen-Str., 52425 Juelich, Germany; 6 Department of Dermatology and Allergology, Ludwig-Maximilians University (LMU), Frauenlobstr. 9-11, 80377 Munich, Germany; 7 Department III of Internal Medicine, Hematology and Oncology, University Hospital Munich, Ludwig-Maximilians University (LMU), 81377 Munich, Germany

**Keywords:** brain metastases, malignant melanoma, robotic stereotactic radiosurgery, SRS, Cyberknife^®^, pseudoprogression, checkpoint inhibition, immunotherapy, radioimmunotherapy

## Abstract

Combination concepts of radiotherapy and immune checkpoint inhibition are currently of high interest. We examined imaging findings, acute toxicity, and local control in patients with melanoma brain metastases receiving programmed death 1 (PD-1) inhibitors and/or robotic stereotactic radiosurgery (SRS). Twenty-six patients treated with SRS alone (*n* = 13; 20 lesions) or in combination with anti-PD-1 therapy (*n* = 13; 28 lesions) were analyzed. Lesion size was evaluated three and six months after SRS using a volumetric assessment based on cranial magnetic resonance imaging (cMRI) and acute toxicity after 12 weeks according to the Common Terminology Criteria for Adverse Events (CTCAE). Local control after six months was comparable (86%, SRS + anti-PD-1, and 80%, SRS). All toxicities reported were less than or equal to grade 2. One metastasis (5%) in the SRS group and six (21%) in the SRS + anti-PD-1 group increased after three months, whereas four (14%) of the six regressed during further follow-ups. This was rated as pseudoprogression (PsP). Three patients (23%) in the SRS + anti-PD-1 group showed characteristics of PsP. Treatment with SRS and anti-PD-1 antibodies can be combined safely in melanoma patients with cerebral metastases. Early volumetric progression of lesions under simultaneous treatment may be related to PsP; thus, the evaluation of combined radioimmunotherapy remains challenging and requires experienced teams.

## 1. Introduction

Immunotherapy and targeted therapy fundamentally changed the treatment of metastasized malignant melanoma (MM) over the last decade [1,2]. Approximately 10 to 40% of MM patients develop brain metastases (BM) during their lifetime [3,4]. Survival time in the metastatic stage is limited to approximately six to ten months [5]. Results from current studies might change strategies in treating BM, since checkpoint inhibition with cytotoxic T-lymphocyte-associated protein 4 (CTLA-4) inhibitors or anti-programmed death 1 (PD-1) inhibitors, as well as targeted therapy, showed remarkable intracerebral response rates [6,7]. Progression of BM in melanoma patients is still a major problem. Current strategies for the management of BM include surgery, whole-brain radiotherapy, and stereotactic radiosurgery (SRS) [8]. Meanwhile, SRS is increasingly used in routine clinical treatment since it shows comparable outcomes associated with less toxicity compared to whole-brain irradiation [9,10,11].

Combination concepts of local radiotherapy with systemic immune checkpoint inhibition are of high interest and currently under prospective investigation due to promising data that arose from retrospective analyses [12,13].

PD-1 is expressed on activated T-cells and mediates inhibitory signals upon binding to its ligand PD-L1, which is expressed on tumor cells and antigen-presenting cells. Deng et al. demonstrated that radiation therapy may increase the expression of programmed death ligand 1 (PD-L1) on tumor cells [14]. A pre-existing immune response against the tumor can, thus, be suppressed by inhibition of T-cell activation using the PD-1/PD-L1 axis, and this is where anti-PD-1 antibodies are able to interfere. So far, data about the outcome and safety of the combined use of SRS and these treatment strategies are limited, and even less is known about concurrently applying checkpoint inhibition and robotic SRS [15].

Pseudoprogression (PsP) is a known imaging observation after SRS for brain lesions [16,17]. It is characterized by a transient increase in contrast-enhancing lesions after radiotherapy, mimicking tumor progression, and resolving or at least stabilizing spontaneously on follow-up imaging without any treatment change. It differs from radiation necrosis in terms of time course and histopathological characteristics [18].

Since the combination of radio- and immunotherapy results in more frequent events of this kind [19], evaluating the response is a relevant challenge after combined treatment. Different strategies have to be developed since PsP should be differentiated from radiation necrosis, and neurological side effects must be individually assessed and evaluated [20].

Therefore, we analyzed how combined local tumor treatment of brain metastases using robotic radiosurgery applied simultaneously with a systemic immune checkpoint blockade affected the magnetic resonance imaging (MRI)-based response after three and six months, acute treatment-related toxicities, and the development of PsP.

## 2. Results

### 2.1. Patient and Lesion Characteristics

Twenty-six MM patients harboring 48 brain metastases (1–5 lesions per patient) were identified. Patient characteristics are presented in Table 1.

We identified 13 patients in each group who either had simultaneous treatment with SRS using Cyberknife^®^ and PD-1 inhibition (SRS + anti-PD-1 group) or single robotic SRS treatment (SRS group). The mean age at the time of robotic SRS application in the SRS + anti-PD-1 group was 54.9 years (54% female patients) versus 61.5 years in the SRS group (38% female patients). The Eastern Cooperative Oncology Group (ECOG) score differed between 0–2 in both groups (SRS + anti-PD-1 group: range 0–2; SRS group: range 0–1). Eight (62%) of the simultaneously treated patients were positive for *BRAF* mutation, five (38%) for *NRAS* mutation, and one for *c-KIT* mutation. In the control group, we found ten (77%) patients to be positive for *BRAF* mutation, one for *NRAS* mutation, and also one for *c-KIT* mutation. Three (23%) patients of the concurrent-treatment group versus five (38%) of the single-treatment group had more than two other metastatic locations outside the brain.

Eight patients of the SRS + anti-PD-1 group had prior systemic treatment, and among those, six patients received ipilimumab in an earlier setting; the last dose was applied at least six months before the robotic SRS treatment. Five patients did not previously receive any systemic treatment. In the SRS group, nine patients were treated with prior systemic treatment; among those, four were ipilimumab-pretreated patients. The SRS group was immunotherapy-free at least two months before and four months after robotic SRS treatment.

The mean time from diagnosis until radiosurgery was 42.2 months (SRS + anti-PD-1) versus 51.1 months (SRS). Differences between both groups were not statistically significant.

A median of two (range: 1–5) lesions per patient was treated in the SRS + anti-PD-1 group and a median of one (range: 1–3) in the SRS group. The median lesion size for the entire cohort was 0.3 cm^3^ (range 0.01–7.4 cm^3^) and amounted to 0.6 cm^3^ (0.1–7.4 cm^3^) in the concurrent treatment group and 0.1 cm^3^ (0.01–2.7 cm^3^) in the control group (*p* = 0.033). Lesion characteristics are presented in Table 2.

### 2.2. Toxicity

Symptoms such as fatigue, headache, nausea, and vertigo of Common Terminology Criteria for Adverse Events (CTCAE) grade 1–2 were observed in both groups. In the SRS + anti-PD-1 group, more symptoms of intracranial pressure (ICP) such as headache, nausea, vertigo, or any combination were reported (eight patients versus five patients in SRS group). Two patients in the SRS + anti-PD-1 group developed enterocolitis accompanied by diarrhea CTCAE grade 2 with positive biopsies in one case. In the SRS group, one of three patients receiving B-Raf and MEK (mitogen-activated protein kinase kinase) inhibitors demonstrated non-specific gastroenterological symptoms CTCAE grade 0–1. Among the simultaneously treated patients, six cases of thyroid disorders were reported. In two of them, they preexisted, and TSH (thyroid-stimulating hormone) levels did not change under treatment. In four patients, previous normal TSH levels fell below the normal range. In the control group, three patients had known thyroid disorders with stable TSH levels under therapy. Overall, there was no statistical significant difference in frequencies of toxicities between the groups; however, newly occurring thyroid disorders were more often seen in the concurrent treatment group. Adverse events are presented in Table 3.

### 2.3. Local Tumor Control and Efficacy Analyses

Regarding the individual lesions after three months, 12 of 28 metastases (43%) of the SRS + anti-PD-1 group decreased in size, while ten (36%) did not change and six (21%) increased compared to the planning cranial MRI (cMRI) scan (baseline). In the SRS group, four of all 20 metastases (20%) showed volumetric regression, while 15 (75%) were stable and one (5%) progressed after three months. This difference in treatment response rate between the SRS + anti-PD-1 and SRS group was statistically significant (*p* = 0.028). After six months, local control (stable or regressed lesions) was satisfactory in both groups: 86% in the SRS + anti-PD-1 group vs. 80% in the SRS alone group.

Furthermore, in the SRS + anti-PD-1 group, we observed a regression of 17 (61%) metastases, seven (25%) were stable, and four (14%) progressed in relation to the baseline cMRI scan after six months. In the SRS group, three (15%) metastases decreased, 13 (65%) were stable, and four (20%) progressed. This difference showed statistical significance (*p* = 0.005). Among the six progressive metastases of the SRS + anti-PD-1 group after three months, four revealed a following regression, one was progressive, and one lesion remained stable. Outcome data are presented in Table 2.

**Figure 1:**
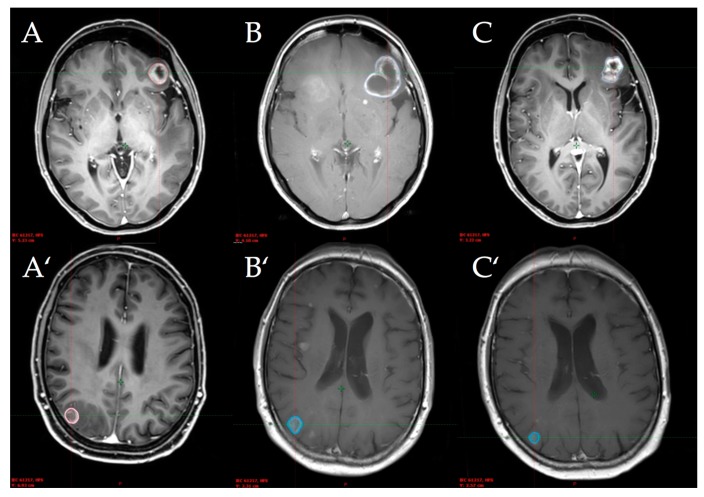
Lesion 1 of simultaneously treated patient No. 1 (top) and lesion 2 of simultaneously treated patient No. 4 (bottom) at baseline (**A**), first follow-up (**B**), and second follow-up (**C**) after stereotactic radiosurgery.

The radiological images of two simultaneously treated patients with an initial increased volume and subsequent regression are shown in Figure 1.

**Figure 2:**
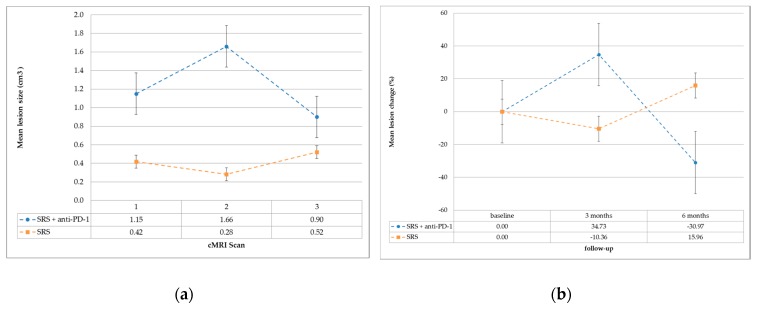
(**a**) Mean lesion size in cm^3^ (standard error bars) at cranial MRI (cMRI) scans 1, 2 and 3. (**b**) Mean lesion change in percentage (standard error bars) before CK (Cyberknife^®^) treatment (baseline), after three and after six months follow-up.

The mean lesion change in both groups is graphically represented in Figure 2 (a: absolute lesion size; b: relative lesion change).

## 3. Discussion

Additionally administered checkpoint inhibition in patients with SRS therapy does not increase treatment-related toxicity and can be applied safely.

In this study we evaluated the strictly simultaneously applied combination treatment of robotic stereotactic radiosurgery using Cyberknife^®^ anti-PD-1 therapy in patients with brain metastases of MM. We retrospectively analyzed this combined treatment in terms of local control and toxicity. Since ipilimumab was approved for the treatment of metastatic melanoma in 2011, most cases of SRS and immunotherapy are based on ipilimumab. The next step in checkpoint inhibition was the approval of anti-PD-1 antibodies in 2014 (e.g., pembrolizumab and nivolumab). The interaction of PD-1/PD-L1 inhibits T-cell activation and cytokine production, and provides an immune escape for cancer cells by turning off cytotoxic T cells [21]. High-dose radiotherapy clinically used in hypo-fractionated regimens or ablative stereotactic radiotherapy is thought to induce pronounced anti-tumor immunity by inducing necrosis, necroptosis, and senescence. However, the data are contradictory and it is not clear which irradiation doses are the “most immunogenic” [22,23,24].

Tumor microenvironment may shift toward anti-cancer immunity by radiation-induced release of damage-associated molecules [25,26]. The abscopal effect is described as the shrinking of distant, non-treated lesions, reported frequently in mice but rarely in humans, and considered the visual evidence for the efficient immune-stimulation by irradiation [24,27,28]. Evidence suggests that irradiation could enhance the effect of immunotherapy or that radiation effects may be intensified by immunotherapy.

We evaluated simultaneously treated patients since radioimmunotherapy was shown to produce better local responses [15,29,30,31]. Several recent studies showed a trend toward improved outcome in terms of improved local control and reduced rates of distant brain failure in patients who received stereotactic radiosurgery in context with anti-PD-1 therapy [32,33,34], and, according to recent literature, the application seems to be safe [35]. We analyzed the feasibility in terms of toxicity. In our cohorts, baseline patients’ characteristics were well balanced (Table 1) and patients were treated by the same multi-disciplinary team using guideline-approved treatment approaches and follow-ups.

This report is a small retrospective study. Lesions in the concurrent treatment group tended to have larger baseline GTVs, and volumes in the control group were relatively small, which made it difficult to detect PsP there since changes in lesion size would also be small.

### 3.1. Response Rate

At the first follow-up after three months, we saw a better local response of all lesions (measuring the GTV via volumetry) in the non-concurrent treatment group. We found 95% of all lesions decreased in volume or were stable (SRS group) versus 79% in the combined group.

However, in the second follow-up after six months, we observed a local response (stable or decreased volume) in 86% of the treated metastases in the SRS + anti-PD-1 group compared to the baseline cMRI scan. In the SRS group, local response was measured in 80% of the lesions.

Since we observed that four out of the six initially enlarging lesions in the SRS + anti-PD-1 group decreased in size after three further months of follow-up, we defined that as pseudoprogression (PsP). Three of the ten stable lesions after SRS + anti-PD-1 treatment had a smaller GTV in the second cMRI scan, while the others remained stable. In these cases, we assumed that PsP was not detected before the first cMRI image was performed. The trend of increasing volume of the treated lesions in the first follow-up and decreasing afterward in patients additionally treated with inhibition of PD-1 is graphically demonstrated as absolute lesion size in Figure 2a and relative lesion change in Figure 2b. Metastases in the SRS only group tended to stay stable or even decrease in volume in the first follow-up, and rather, increase afterward. This supports the theory that tumor tissue reacts in a different manner to irradiation when the patient is concurrently treated with checkpoint inhibitors. Taken together, we assume that PsP may occur even more frequently than in 23% of simultaneously treated patients. This has to be shown in larger cohorts of patients.

Overall, only a limited number of studies concerning PsP exist, and most of them are case studies or address gliomas [20,36]. In our study, we outlined PsP in four (14%) metastases of three (23%) different patients in the SRS + anti-PD-1 group versus no cases of PsP in the SRS group. PsP in primary and secondary brain tumors are known to be observed at any time from weeks up to several months after irradiation, mostly not accompanied by clinical symptoms, but merely showing enhanced lesions on MRI scans; nonetheless, some patients can present complications [37].

Many different reasons for PsP are under consideration, including its pathophysiology and associated molecular changes [38,39]. Biopsies at the time of lesion progression could help clarify the mechanisms leading to real or pseudoprogression. Rauch et al. report that lesions regrown after SRS show leukoencephalopathic changes and inflammatory changes, which contained dispersed CD3^+^ T lymphocytes within the parenchyma and vascular walls [38]. Patel et al. consider that patients with a strongly activated immune system might respond to high irradiation doses with a strong inflammatory response at the irradiated site, resulting in the growth of the lesion [16].

The mechanisms of PsP remain unknown, but literature suggests that it is likely due to a combination of tumor necrosis, edema, and secondary inflammation resulting from DNA and cell damage, release of cell fragments acting as neo-antigens, and migration of antigen-presenting cells and immune cells [17]. The induction of PsP might be related to different interacting factors.

Since PsP of brain lesions was also observed after checkpoint inhibition alone with similar histopathological findings (inflammatory infiltrate with activated microglial cells and scattered CD8^+^ T cells) [39], it stands to reason that irradiation plus checkpoint inhibition enhances the probability of PsP appearing. To distinguish reliably between PsP and actual progress, a sufficiently long follow-up period must be observed and biopsies of growing lesions should be taken to detect the underlying mechanisms and complex reactions of PsP.

### 3.2. Toxicity

Three months after robotic SRS treatment regarding the simultaneously treated (SRS + anti-PD-1) patients, we observed symptoms of immune toxicities such as colitis and thyroid disorder, as expected since they were reported by several other groups [2,40,41]. Two patients who suffered from colitis also developed thyroid dysfunction, and both patients showed a good clinical response. Looking at other studies, this supports the theory that patients with a tendency to autoimmunity may be more likely to benefit from anti-PD-1 antibody therapy [42].

The overall rates of acute toxicity were higher in the SRS + anti-PD-1 group, but neither exceeded CTCAE grade 2 adverse events, nor reached statistical significance. Those patients also developed more symptoms of intracranial pressure such as headache and/or nausea and/or vertigo, which was unexpected but presumably due to the greater median lesion size in this group (Table 2). This might also be rated as symptoms of PsP.

## 4. Materials and Methods

### 4.1. Patients

Data were analyzed retrospectively from an institutional database of malignant melanoma patients treated with radiotherapy and immunotherapy. The analysis was approved by an Institutional Review Board. We included patients diagnosed with brain metastases and treated with single-fraction robotic SRS between August 2011 and September 2016. In total, 26 patients with 48 lesions were analyzed. Data collected included baseline demographics, Eastern Cooperative Oncology Group performance status (ECOG), mutational status (*BRAF*/*NRAS*/*c-KIT*), number of brain lesions at the planning cranial magnetic resonance imaging (cMRI) scan, prior systemic treatments, PD-1 inhibitor treatment (type, applied cycles), and acute side effects according to the Common Terminology Criteria for Adverse Events (CTCAE) v4.0.

### 4.2. Immunotherapy

Patients were considered to have received simultaneous treatment of PD-1 inhibitors with robotic SRS if the first dose was administered at least one week before robotic SRS treatment and was continued for at least six weeks after irradiation (*n* = 13). Patients who had robotic SRS at least three months after the last cycle of immunotherapy or other treatments, and at least six months before the first cycle were considered to have received non-simultaneous treatment (*n* = 13). Patients with simultaneous treatment received at least three cycles of intravenous anti-PD-1 therapy with either pembrolizumab at a dose of 2 mg/kg every three weeks (*n* = 10) or nivolumab at a dose of 3 mg/kg every two weeks (*n* = 3).

### 4.3. Robotic Stereotactic Radiosurgery (SRS) with Cyberknife^®^

A high-resolution contrast-enhanced computed tomography (CT) was co-registered and fused with a high-resolution contrast-enhanced cMRI with T1- and T2-weighted images (Philips—Ingenia 3.0 Tesla MR-system) for Cyberknife^®^ treatment planning. Treatment planning was carried out by a team of experienced physicians and physicists. The treatment planning software, Multiplan v4.5 (Accuray Inc., Sunnyvale, CA, USA), was used for contouring the gross tumor volume (GTV) and the organs at risk (OARs). Robotic SRS was performed with a single fraction of 18–22 Gy (=65% isodose) for one to five metastases per patient. Patient immobilization was achieved by a thermoplastic mask fixed to the Cyberknife^®^ treatment table. To prevent brain edema, cortisone (dexamethasone 4 mg) was administered on the day of treatment and the following day.

### 4.4. Follow-Up

Patients were followed with regular clinical examinations and cMRI imaging in three-month intervals. Lesions (*n* = 48) were analyzed via volumetry of the treatment planning cMRI scan, as well as the first and second follow-up cMRI scan. The gross tumor volume (GTV) was outlined independently by two radio-oncologists (C.B. and M.T.-N.) in every T1-weighed cMRI scan using the radiotherapy planning software (Eclipse version 13.6, Varian) and measured in cm^3^. According to the response assessment criteria for brain metastases proposed by the RANO-BM (Response Assessment in Neuro-Oncology Brain Metastases) working group [43], we defined volumetric reduction or increase if a single lesion demonstrated a 20% or more volumetric change and an absolute change of 0.1 cm^3^. A lesion was considered to be locally controlled if the GTV decreased, did not change, or increased within these limits. Primary endpoints were local control after three and six months and toxicity within 90 days after robotic SRS treatment.

PsP as described above was assumed in cases of a ≥20% volumetric increase of all lesions in the first follow-up relative to the baseline cMRI scan, followed by a ≥20% volumetric decrease of all lesions in the second follow-up relative to the first follow-up. Acute side effects were evaluated by analyzing medical reports after application of robotic SRS.

### 4.5. Statistical Analyses

All statistical analyses were performed using SPSS v.21 (IBM Corp., Armonk, NY, USA). Baseline and lesion characteristics, as well as treatment-related toxicities, were compared using the *t*-test for independent continuous variables and Pearson’s chi-square test or Fisher’s exact test for categorical variables where appropriate. In any case, *p*-values < 0.05 were considered significant.

## 5. Conclusions

Concomitant SRS and checkpoint inhibition does not increase therapy-related toxicity and can be safely administered. Based on our results, which agree with the literature, more than 20% of patients after robotic SRS in combination with anti-PD-1 antibodies may develop volumetric progression in the context of PsP. The recognition of tumor progression versus PsP or radiation necrosis after SRS [30,44] and management of symptomatic patients is essential. Changes in MRI scans can still be seen years after SRS [16,17,45,46]. In case of uncertainty, amino-acid PET scans or even biopsies may be necessary to distinguish PsP from true progression to reduce the risk of inadequate treatment [47,48].

Although immune-related response criteria were developed [43,49] to better assess response after immunotherapy, the need for specially trained teams to evaluate radiological imaging of immunotherapy-treated patients is increasing.

Additional studies with larger patient cohorts and long-term follow-ups are required to better understand this complex field of interactions. In the meantime, the increasing implementation and positive results of several studies applying peri-SRS immune checkpoint inhibitors make our study an important contribution to demonstrate the safety of combining these therapies and to be aware of different possible treatment responses.

## Figures and Tables

**Table 1:** Baseline demographics.

Characteristic		All Patients	SRS + Anti-PD-1	SRS	*p*-Value
Patients n		26	13	13	
Age (y) at SRS-treatment	median (IQR)	57 (32.5)	52 (27)	64 (31)	0.314
	range	33–84	36–81	33–84	
Gender	male (% of pts.)	14 (54)	6 (46)	8 (62)	0.431
	female (% of pts.)	12 (46)	7 (54)	5 (38)	
ECOG	0 (% of pts.)	4 (15)	2 (15)	2 (15)	0.18
	1 (% of pts.)	18 (69)	7 (54)	11 (85)	
	2 (% of pts.)	4 (15)	4 (31)	0	
Mutations	*BRAF* (% of pts.)	18 (69)	8 (62)	10 (77)	0.317
	*NRAS* (% of pts.)	6 (23)	5 (38)	1 (8)	0.155
	*c-KIT* (% of pts.)	2 (8)	1 (8)	1 (8)	1
Metastases	1 other location than brain (% of pts.)	5 (19)	2 (15)	3 (23)	
	2 other locations than brain (% of pts.)	13 (50)	8 (62)	5 (38)	0.584
	>2 other locations than brain (% of pts.)	8 (31)	3 (23)	5 (38)	
Prior systemic therapy	B-Raf inhibitor	11	3	8	
	MEK inhibitor	5	3	2	
	TK-/RTK-inhibitor	2	0	2	
	chemotherapy	10	4	6	
	interferon	2	2	0	
	ipilimumab	10	6	4	
	none	9	5	4	
Type of PD-1 inhibitor	pembrolizumab (% of pts.)	10 (38)	10 (77)	-	
	nivolumab (% of pts.)	2(12)	3 (23)	-	
Interval Diagnosis SRS (m)	median (IQR)	33.5 (33.3)	29 (29)	45 (45)	0.724
	range	1–204	1–129	4–204	

Baseline demographics of all patients, and patients separated into simultaneously treated group (SRS + anti-PD-1) and single treatment group (SRS). Statistical analyses show differences in frequencies between the two groups. Differences were regarded statistically significant if *p* < 0.05. SRS = stereotactic radiosurgery; PD-1 = programmed death 1; ECOG = Eastern Cooperative Oncology Group score; y = years; m = months; pts. = patients; IQR = interquartile range; (R)TK = (receptor) tyrosine kinase; B-Raf = protein encoded by gene BRAF; MEK = mitogen-activated protein kinase kinase.

**Table 2:** Lesion characteristics and outcome.

Characteristic		All Patients	SRS + Anti-PD-1	SRS	*p*-Value
Patients n (%)		26	13 (50)	13 (50)	
Lesions n (%)		48	28 (58)	20 (42)	
Lesions treated per patient	median (IQR)	1.5 (2)	2 (2)	1 (1)	0.555
	range	1–5	1–5	1–3	
GTV in cm^3^—baseline	median (IQR)	0.3 (0.95)	0.55 (1.23)	0.1 (0.38)	0.033
	range	0.01–7.4	0.01–7.4	0.01–2.7	
Dose in Gy, 65% isodose level	range	18–22	18–20	18–22	1
**Outcome**					
GTV in cm^3^—1st follow-up	median (IQR)	0.2 (0.57)	0.2 (1.14)	0.2 (0.34)	0.561
	range	0.0–17.8	0.0–17.8	0.0–1.5	
1st follow-up vs. baseline	regression (% of les.)	16 (33)	12 (43)	4 (20)	0.028
	stable (% of les.)	25 (52)	10 (36)	15 (75)	
	progression (% of les.)	7 (15)	6 (21)	1 (5)	
GTV in cm^3^—2nd follow-up	median (IQR)	0.06 (0.4)	0.06 (0.37)	0.06 (0.93)	0.551
	range	0.0–6.4	0.0–6.4	0.0–2.4	
2nd follow-up vs. baseline	regression (% of les.)	20 (42)	17 (61)	3 (15)	0.005
	stable (% of les.)	20 (42)	7 (25)	13 (65)	
	progression (% of les.)	8 (17)	4 (14)	4 (20)	
2nd follow-up	pseudoprogression (% of les.)	4 (8)	4 (14)	0	0.13

Lesion characteristics and outcome of all patients, and patients separated into simultaneously treated group (SRS + anti-PD-1) and single treatment group (SRS). Statistical analyses show differences in frequencies between the two groups. Differences were regarded statistically significant if *p* < 0.05. GTV = gross tumor volume; les. = lesions.

**Table 3:** Adverse events.

Characteristic		SRS + Anti-PD1 (*n* = 13)			SRS (*n* = 13)			*p*-Value
		Grade 0–1	Grade 2	All Grades	Grade 0–1	Grade 2	All Grades	
Any Toxicity (pts.)				10			7	0.411
Symptoms of ICP (pts.)				8			5	0.239
	headache	4	2	(6)	3	0	(3)	0.352
	nausea	1	1	(2)	1	0	(1)	1
	vertigo	3	1	(4)	2	0	(2)	0.645
Fatigue (pts.)		3	1	(4)	2	1	(3)	1
Thyroid disorder (pts.)				6			3	0.411
	new	2	2	(4)	0	0	(0)	0.096
	known	0	2	(2)	2	1	(3)	0.593
Gastroenterological symptoms (pts.)				2			3	1
	colitis	0	2	(2)	0	0	(0)	0.48
	unspecific	0	0	(0)	3	0	(3)	0.22

Adverse events and number of patients who developed any toxicity during the first three months follow-up. Statistical analyses were calculated using only numbers without brackets. Statistical analyses show differences in frequencies between the two groups. Differences were regarded statistically significant if *p* < 0.05. ICP = intracranial pressure.

## Abbreviations

SRS	stereotactic radiosurgery
PD-1	programmed death 1
MM	malignant melanoma
BM	brain metastases
PD-L1	programmed death ligand 1
PsP	pseudoprogression
ECOG	Eastern Cooperative Oncology Group performance status
cMRI	cranial magnetic resonance imaging
CTCAE	Common Terminology Criteria for Adverse Events
CT	computed tomography
GTV	gross tumor volume
OAR	organs at risk
ICP	intracranial pressure
